# Supplementary material for: Chronic Intermittent Hypoxia during Sleep Causes Browning of Interscapular Adipose Tissue Accompanied by Local Insulin Resistance in Mice
Source: Int J Mol Sci. 2022 Dec 7;23(24):15462. doi: 10.3390/ijms232415462 (PMC9779339; doi:10.3390/ijms232415462)
Supplement: Supplementary file 1 [file ijms-23-15462-s001.zip › ijms-1960229-supplementary.pdf]

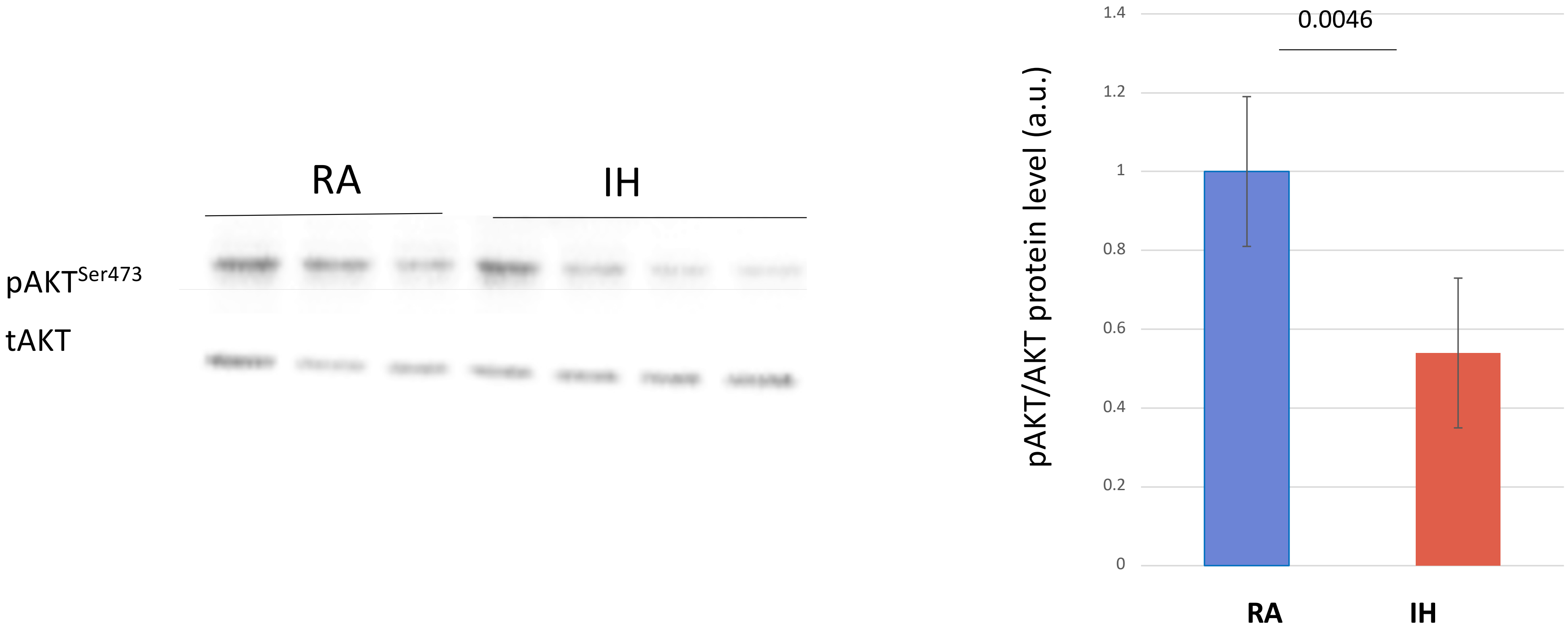

Supplemental Figure S1: Western Blot analysis of phosphorylated AKT(Ser473) protein levels normalized to total Akt in skeletal muscle following I.P insulin stimulation. (n=3-4)
